# Establishment and characterization of patient-derived mucinous gastric cancer organoids with faithful histopathological features and reproducible drug response patterns

**DOI:** 10.3389/fonc.2026.1905751

**Published:** 2026-09-17

**Authors:** Haobin Hou, Sufen Fang, Wei Chen, Yulong He, Xuefu Zhou, Tengfei Hao

**Affiliations:** Digestive Diseases Center, The Seventh Affiliated Hospital of Sun Yat-sen University, Shenzhen, China

**Keywords:** chemoresistance, high-throughput drug sensitivity assays, mucinous gastric cancer, mucus secretion, patient-derived organoids

## Abstract

**Background:**

Mucinous gastric cancer (MGC) is a rare subtype of gastric cancer (GC) characterized by distinct histological features and clinical behavior. However, the lack of specific *in vitro* models for MGC has hindered progress in understanding its carcinogenic mechanisms and developing effective therapeutic strategies.

**Methods:**

Patient-derived MGC organoids were established from paired primary tumor (MGC-T) and synchronous peritoneal metastatic nodule (MGC-ND) tissues obtained from a patient with MGC. To validate the generalizability of the findings, two additional independent MGC organoids (MGC2 and MGC3) and six non-MGC gastric cancer organoids were established in parallel under identical three-dimensional culture conditions. Histological and immunohistochemical analyses were performed to evaluate the fidelity of organoid recapitulation of parental tumor features. High-throughput drug sensitivity screening was conducted using a panel of 11 chemotherapeutic agents. Furthermore, organoid-based drug responses were correlated with the clinical outcomes of eight patients who received oxaliplatin-based adjuvant chemotherapy.

**Results:**

All MGC organoids faithfully maintained the histological architecture, mucus secretion capability, and biomarker profiles of the parental tumors over serial passages. High-throughput drug sensitivity screening revealed consistent resistance to 5-fluorouracil and oxaliplatin, and marked sensitivity to taxane-based agents, across the MGC panel. Preliminary clinical correlation in eight patients showed 87.5% concordance between organoid-based predictions and actual chemotherapy outcomes.

**Conclusion:**

We established and characterized patient-derived MGC organoids from three patients, demonstrating faithful histological and molecular preservation, dynamic mucus secretion, and reproducible drug sensitivity pattern. Preliminary clinical correlation in a small cohort showed promising concordance. These proof-of-concept findings establish a platform for future precision oncology research in this rare gastric cancer subtype, pending larger prospective validation.

## 1 Introduction

Gastric cancer (GC) ranks as the fifth most common cancer and fifth leading cause of cancer-related deaths globally (1). According to WHO classification, gastric adenocarcinoma can be morphologically categorized into several subtypes, including papillary adenocarcinoma, tubular adenocarcinoma, mucinous adenocarcinoma, poorly cohesive carcinoma (including signet ring cell carcinoma), mixed adenocarcinoma and other rare variants (2). As a distinct pathological subtype, mucinous gastric cancer (MGC) accounts for 2.8-6.6% of all gastric adenocarcinomas (3) and is characterized by significant mucin secretion. The WHO classification defines MGC as gastric adenocarcinoma containing over 50% extracellular mucin within tumors (4).

Microscopically, MGC exhibits two distinct growth patterns: glandular/tubular structures with interstitial mucin, or floating tumor cells in mucin lakes (5). The cancer cells display irregular shapes, variable sizes, and increased mitotic activity, often accompanied by nuclear degeneration, nuclear fragmentation, and necrosis. The mucin composition in MGC is characterized by unique features that distinguish it from normal gastric tissue. While normal gastric mucosa primarily expresses MUC1, MUC5AC, and MUC6, MGC shows significantly elevated MUC2 expression, found in 95.5% of MGC cases compared to only 33.4% in non-mucinous gastric cancer (6). Moreover, MGC predominantly exhibits an intestinal mucinous phenotype, accounting for 60.9% of cases, followed by a mixed phenotype at 34.6%, while the gastric mucinous phenotype remains rare, comprising only 2.3% of cases (7). This distinct mucin expression pattern suggests fundamental differences in the biological behavior of MGC compared to other gastric cancer subtypes (8).

The pathogenesis of MGC remains to be fully elucidated. The classic model of gastric carcinogenesis involves a sequential progression from chronic gastritis to intestinal metaplasia, then to dysplasia, and finally to carcinoma (9). While intestinal metaplasia is recognized as an intermediate stage in GC development (10, 11), characterized by decreased gastric-type mucins (MUC1, MUC5AC, MUC6) and increased MUC2 expression (12), the relatively low prevalence of MGC compared to intestinal-type GC suggests the involvement of additional mechanisms. However, research into these mechanisms has been hampered by the limited availability of appropriate *in vitro* models that faithfully recapitulate the unique biological features of MGC. Understanding these mechanisms is crucial for developing effective therapeutic strategies.

Research into MGC has been hampered by limited availability of appropriate preclinical models that faithfully recapitulate the unique biological features of the disease. To address these limitations, we have successfully established a panel of patient-derived MGC organoids and comprehensively characterized their histopathological and molecular fidelity. Leveraging these models, we performed high-throughput drug sensitivity screening against a panel of clinically relevant agents and further validated the organoid-based predictions against actual patient responses. Our findings suggest a reproducible drug sensitivity pattern across the examined MGC organoids—marked by resistance to standard first-line regimens but heightened sensitivity to taxanes—and indicate that patient-derived organoids may serve as a valuable platform for guiding individualized treatment strategies in this understudied gastric cancer subtype.

## 2 Materials and methods

### 2.1 Patient and samples

Human GC tissue samples were collected from GC surgery patients at The Seventh Affiliated Hospital of Sun Yat-sen University after obtaining written informed consent. The study was approved by the Medical Ethics Committee of The Seventh Affiliated Hospital of Sun Yat-sen University (approval number: KY-2022-039-02) and adhered to the principles of the Declaration of Helsinki. Paired tumor tissue samples were obtained from the primary gastric lesion and a synchronous peritoneal metastatic nodule for organoid culture establishment. In addition, tumor tissues from two other patients with histologically confirmed MGC and six patients with non-MGC were collected under the same protocol for organoid culture establishment and comparative analysis.

### 2.2 Organoid culture

Fresh tumor tissue was obtained immediately after surgical resection and placed in ice-cold DMEM (Gibco, 11965-092) at 4 °C, and transported to the laboratory within 30 minutes. Upon arrival, the tissue was washed three times with ice-cold PBS (Gibco, 10010-023) containing 5% penicillin-streptomycin (Gibco, 15140-122), and minced into approximately 1–2 mm³ fragments using sterile surgical scissors. The minced fragments were transferred to a 15 mL centrifuge tube and washed repeatedly with PBS containing 1% penicillin-streptomycin until the supernatant became clear.

For tissue digestion, the fragments were resuspended in digestion buffer consisting of DMEM (Gibco, 11965-092) supplemented with 1 mg/mL collagenase V (Sigma-Aldrich, C9263), 0.1 mg/mL DNase I (Roche, 11284932001), and 10 µM Y-27632 (AbMole, M1817). To overcome the challenges posed by the mucin-rich extracellular matrix characteristic of MGC, the digestion buffer was further supplemented with 0.5 mg/mL hyaluronidase (Sigma-Aldrich, H3506). The mixture was incubated at 37 °C for 1–2 hours on an orbital shaker at 100 rpm, with intermittent pipetting every 20 minutes to facilitate dissociation. For tumors with substantial mucin content, the digestion time was extended up to 2.5 hours.

The digested suspension was filtered through a 100 µm cell strainer (Falcon, 352360) to remove undigested debris, and the filtrate was centrifuged at 300 ×g for 5 minutes at 4 °C. The cell pellet was washed twice with ice-cold PBS containing 1% FBS to remove residual enzymes. The resulting cell pellet was resuspended in ice-cold Matrigel (Corning, 356231) at a density of 2 × 10^4^ cells per 40 µL of Matrigel, and plated as 40 µL domes into pre-warmed 24-well plates (Corning, 3524). The plates were incubated at 37 °C for 30 minutes to allow Matrigel polymerization.

After polymerization, 500 µL of organoid-specific medium was added to each well. The complete medium consisted of Advanced DMEM/F12 (Gibco, 12634-028) supplemented with 1×B-27 (Gibco, 12587-010), 0.5 mM N-acetylcysteine (Sigma-Aldrich, A9165), 50 ng/mL recombinant human EGF (Peprotech, AF-100-15), 100 ng/mL FGF10 (Peprotech, 100-26), 100 ng/mL Noggin (Peprotech, 250-38), 500 ng/mL R-Spondin 1 (R&D Systems, 4645-RS), 100 ng/mL Wnt3a (R&D Systems, 5036-WN), 5 nM Gastrin I (Tocris, 3006), 0.25 µM A83-01 (Tocris, 2939), 10 µM Y-27632 (AbMole, M1817) (added only for the first 3 days after seeding), and 100 µg/mL Primocin (Invivogen, ant-pm-1). To selectively inhibit normal epithelial contamination, Wnt3a was withdrawn and R-Spondin 1 was reduced to 100 ng/mL from passage 2 onward.

The culture medium was replaced every 2–3 days with fresh pre-warmed medium. Primary organoids typically emerged within 7–14 days after seeding. Organoids were passaged at a 1:3 to 1:4 ratio every 7–10 days when they reached a diameter of 100-200 µm. For passaging, the Matrigel domes were mechanically disrupted by pipetting with ice-cold PBS, and organoids were collected by centrifugation at 200 ×g for 5 minutes. The organoid pellet was dissociated with TrypLE Express (Gibco, 12605-028) at 37°C for 3–5 minutes with gentle pipetting, followed by neutralization with Advanced DMEM/F12 containing 10% FBS. After centrifugation, the cell pellet was resuspended in fresh Matrigel and replated as described above. All experiments were performed using organoids at passages 3–8.

For cryopreservation, organoids were harvested and dissociated as described above, then resuspended in 90% FBS + 10% DMSO (Sigma-Aldrich, D2650) at a density of approximately 5 × 10^5^ cells per vial. The vials were placed in a Mr. Frosty freezing container (Thermo Scientific, 5100-0001) and stored at −80 °C overnight, then transferred to liquid nitrogen for long-term storage. For recovery, frozen vials were thawed rapidly in a 37 °C water bath, washed once with pre-warmed medium, and replated in Matrigel as described above. The recovery success rate was consistently >80% across all established lines.

### 2.3 Immunohistochemical staining

Surgical tumor specimens were fixed in 10% neutral buffered formalin for 24 hours, then dehydrated through a graded series of ethanol (75%, 85%, 95%, 100%) and embedded in paraffin. Organoid samples were similarly fixed and embedded. Sections of 5 μm thickness were cut and stained with hematoxylin and eosin (HE) using standard procedures. For IHC, after deparaffinization, sections underwent heat-mediated antigen retrieval in either 10 mM citrate buffer or Tri-EDTA, and were then treated with 1.6% hydrogen peroxide in methanol to block endogenous peroxidase activity. Primary antibodies were applied at the following dilutions: anti-Ki67 (1:4000, Proteintech, 27309-1-AP), anti-TP53 (1:250, Proteintech, 10442-1-AP), anti-pan-CK (1:1500, Proteintech, 26411-1-AP), anti-E-cadherin (1:5000, Proteintech, 20874-1-AP), anti-CDX2 (1:500, Proteintech, 82659-1-RR), anti-MUC5AC (1:100, Abcam, ab3649), and anti-MUC2 (1:1000, Proteintech, 27675-1-AP). All primary antibodies were incubated overnight at 4°C. After washing, sections were incubated with horseradish peroxidase (HRP)-conjugated secondary antibodies (1:200, anti-mouse or anti-rabbit IgG, Cell Signaling Technology, 7076S/7074S) for 1 hour at room temperature. Immunoreactivity was visualized using 3,3′-diaminobenzidine (DAB) substrate (Agilent/Dako, K3468), and sections were counterstained with Mayer’s hematoxylin (Sigma-Aldrich, MH58016). Isotype-matched IgG (same concentration as the primary antibody, Abcam, ab37415) served as the negative control, and known positive tissue sections were included as positive controls for each antibody. All histological comparisons between organoid sections and their corresponding primary tumor specimens were independently reviewed by two board-certified pathologists with expertise in gastrointestinal pathology. The reviewers were blinded to the sample identity during the evaluation, and any discrepancies were resolved through discussion to reach a consensus.

### 2.4 Immunofluorescent staining

Tissue sections (5 μm) were deparaffinized, rehydrated, and subjected to heat-induced antigen retrieval using 10 mM sodium citrate buffer (pH 6.0) (Solarbio, C1032) or Tris-EDTA buffer (pH 9.0) (Solarbio, C1040). After blocking with PBS (Gibco, 10010-023) containing 5% bovine serum albumin (BSA; Sigma-Aldrich, A9418) and 0.2% Triton™ X-100 (Sigma-Aldrich, T8787) for 1 hour at room temperature, sections were incubated with primary antibodies (diluted in blocking buffer) overnight at 4 °C. After three washes with PBS, sections were incubated with fluorescein-conjugated secondary antibodies (Alexa Fluor™ 488/555/647, 1:500, Thermo Fisher Scientific, A32723/A32727/A32733) for 1 hour at room temperature in the dark. Nuclei were counterstained with DAPI (4′,6-diamidino-2-phenylindole, 0.1 μg/mL; Thermo Fisher Scientific, D3571) for 10 minutes. Finally, sections were mounted with anti-fade mounting medium (Beyotime, P0126) and imaged using a Zeiss LSM 780 confocal microscope (Carl Zeiss, Germany).

### 2.5 Alcian blue-periodic acid schiff staining

Organoids and tumor tissues were fixed in 10% neutral buffered formalin for 16–20 hours, then embedded in paraffin, sectioned at 5 μm, baked, and deparaffinized. For AB-PAS staining, the sections were first stained with Alcian Blue solution (pH 2.5, Solarbio, G1285-1) for 5–10 minutes and rinsed with distilled water. Next, they were treated with Periodic Acid solution (Solarbio, G1285-2) for 5 minutes, rinsed, and then incubated with Schiff’s reagent (Solarbio, G1285-3) for 5–10 minutes, followed by a final distilled water rinse. The samples were subsequently dehydrated through graded ethanol, cleared in xylene, mounted with neutral resin, and examined under a light microscope.

### 2.6 Live-cell imaging

For real-time observation of mucin secretion dynamics, MGC-ND organoids were dissociated using TrypLE at 37 °C for 15 minutes until single cells or small cell clusters were observed under microscopy. After centrifugation and removal of the supernatant, the cell pellet was resuspended in culture medium supplemented with 5% Matrigel and seeded into a 24-well plate at a low density (~1,000 cell clusters per well). The plate was then placed in a Lionheart FX automated live-cell imaging system (BioTek, Winooski, VT, USA) equipped with a temperature-controlled chamber (37 °C, 5% CO_2_). Images were captured at 5-minute intervals over a 48 hours period.

### 2.7 Drug screening

A 384-well white flat-bottom plate (Corning, 3764) was pre-dosed with an FDA-approved drug library at 10 serial concentrations (3-fold dilution series) in triplicate (technical replicates) using an acoustic liquid handler (Echo^®^ 650, Beckman Coulter). 0.1% DMSO (Sigma-Aldrich, D8418) served as the negative control (0% inhibition) and 4 µM MG132 (Selleck Chemicals, S2619) as the positive control (100% inhibition). Organoids at passages 3–6 were dissociated using TrypLE™ Express (Gibco, 12605-028) at 37 °C for 10 minutes, and the resulting single-cell suspension was collected. A mixture containing 1 × 10^4^ cells and Matrigel^®^ (Corning, 356231) was dispensed into each drug-pre-loaded well using an automated dispenser (MultiFlo™ FX, Agilent/BioTek). After 72 hours of culture, cell viability was assessed using the CellTiter-Glo^®^ 2.0 Assay (Promega, G9242) (13). Luminescence was recorded as relative light units (RLU) on a Spark^®^ microplate reader (Tecan).

RLU values were normalized to DMSO controls (set as 100% viability), and dose–response curves were fitted using a four-parameter logistic (4-PL) model with a variable slope in GraphPad Prism 9.0 (Dotmatics) using the equation: Y = Bottom + (Top − Bottom)/(1 + 10^ ((LogIC50 − X) × HillSlope)). No constraints were imposed on Top or Bottom to allow free fitting to the empirical data. The IC50 values were calculated as the drug concentration required to reduce cell viability by 50%, and are reported with 95% confidence intervals (CIs). The goodness-of-fit was assessed by R² values, and assay quality was monitored by the Z′-factor calculated from positive and negative controls, with a threshold of Z′ > 0.5 considered acceptable. Each experiment was performed in three independent biological replicates, with three technical replicates (wells) per concentration in each experiment. Data from all independent experiments were pooled for final analysis.

### 2.8 Clinical correlation analysis

For patients who received oxaliplatin-based adjuvant chemotherapy after gastrectomy, clinical outcomes were monitored by computed tomography every 3–6 months. Progression-free survival was defined as the time from surgery to radiological recurrence or death from any cause. Patients who experienced radiological recurrence within 6 months after surgery were classified as poor responders, consistent with the typical window for early recurrence detection in gastric cancer patients receiving adjuvant chemotherapy. For organoid-based prediction, combination drug sensitivity assays using oxaliplatin plus 5-fluorouracil were performed, and the area under the dose-response curve (AUC) was calculated. An AUC ≥ 50% was defined as resistant, and AUC < 50% as sensitive. Concordance between organoid prediction and clinical outcome was evaluated by direct comparison of sensitivity/resistance classification.

## 3 Results

### 3.1 MGC patient management and workflow for MGC organoids establishment, cultivation, and characterization

A 73-year-old male patient presented with dysphagia. Gastroscopy revealed an ulcerative lesion extending from the cardia to the fundus (**Figure 1A**), and biopsy confirmed MGC with a minor signet ring cell component. CT imaging demonstrated a mass near the cardia with serosal involvement, along with slightly enlarged regional lymph nodes, consistent with a clinical stage of cT4aN1M0 (**Figure 1B**). The patient subsequently underwent a radical total gastrectomy with D2 lymph node dissection (**Figure 1C**). Intraoperatively, a peritoneal metastatic nodule was also identified and resected. Postoperative pathological examination revealed that the tumor was composed of approximately 60% mucinous adenocarcinoma and 40% moderately differentiated tubular adenocarcinoma, with scattered signet ring cells identified within the mucinous component. The tumor invaded the subserosa of the stomach and extended into the superficial muscularis of the esophagus, with 4 of 72 resected lymph nodes harboring metastases. According to the 8th edition of the American Joint Committee on Cancer TNM staging system (14), the final pathological stage was pT3N2M1. The patient completed four cycles of adjuvant chemotherapy with the SOX regimen (oxaliplatin plus S-1) but was subsequently lost to follow-up. The clinical course of this case is summarized chronologically in **Figure 1D**.

**Figure 1:**
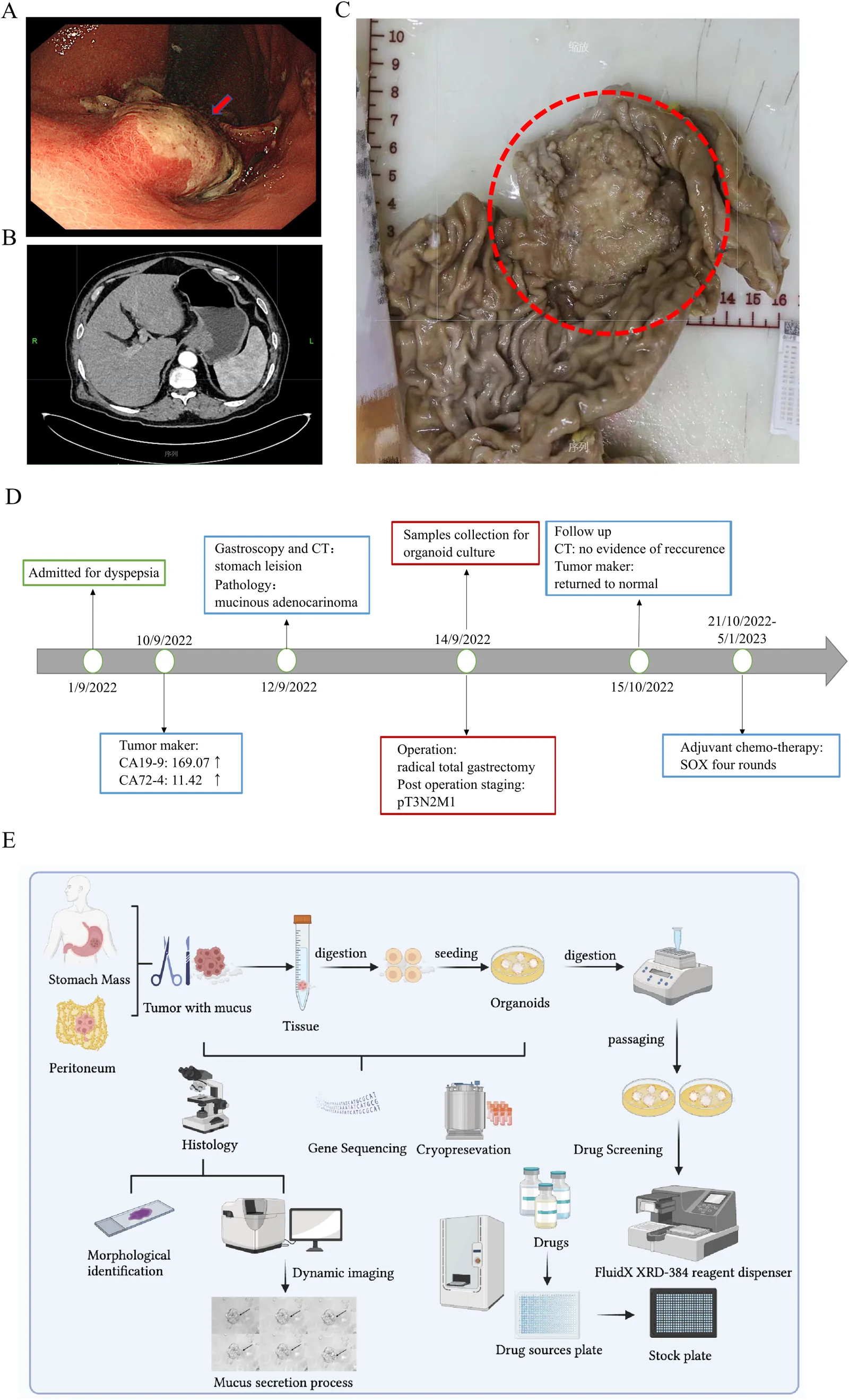
MGC Patient Management and Workflow for MGC Organoids Establishment, Cultivation, and Characterization. **(A)** Gastroscopy revealing a protruding ulcerative lesion extending from the cardia to the fundus. **(B)** CT imaging demonstrating a mass near the cardia with serosal involvement. **(C)** Macroscopic view of the resected stomach specimen, with the mucinous tumor mass highlighted by a red circle. **(D)** Chronological summary of the patient’s clinical course. **(E)** Schematic workflow for organoid establishment, cultivation, and downstream analyses.

Following surgery, tumor tissue from the primary site and the peritoneal metastatic nodule were collected for organoid culture establishment. The organoid derived from the primary tumor was designated MGC-T, while that from the peritoneal metastasis was labeled MGC-ND. After approximately one week of primary culture, both organoids reached sufficient growth for subculturing. At each passage, growth was monitored using optical microscopy. Organoids were allocated to multiple downstream analyses: high-throughput drug sensitivity screening, cryopreservation, and histological processing (fixation, embedding, and sectioning). The experimental workflow is illustrated in **Figure 1E**.

### 3.2 Mucin secretion characteristics and dynamics in MGC organoids

After stable passage was established, we examined the MGC organoids microscopically. In the traditional three-dimensional culture system with Matrigel, single cells initially appeared suspended within the matrix on day one post-digestion. By day four, these cells had formed aggregates with evident luminal structures, and by day seven, fully differentiated organoids were observed suspended in the culture medium, displaying either luminal structures or solid spheroids (**Figure 2A**). Upon serial passaging, both MGC-T and MGC-ND organoids exhibited stable growth and could be routinely passaged at a 1:3 to 1:4 ratio every 7–10 days. The population doubling time was estimated to be approximately 72 hours during the exponential growth phase (passages 3–8) (**Figure 2B**). To evaluate their growth potential without Matrigel, we cultured the organoids under 2D conditions. By day four, the organoids began to aggregate, with some adhering to the culture plate. By day seven, they had formed elliptical clusters with extended cellular protrusions anchoring to the plate. Unlike gastric cancer cell lines, these organoids did not proliferate to cover the entire plate but instead formed stable clusters that adhered to the surface and exhibited limited growth potential (**Figure 2A**).

**Figure 2:**
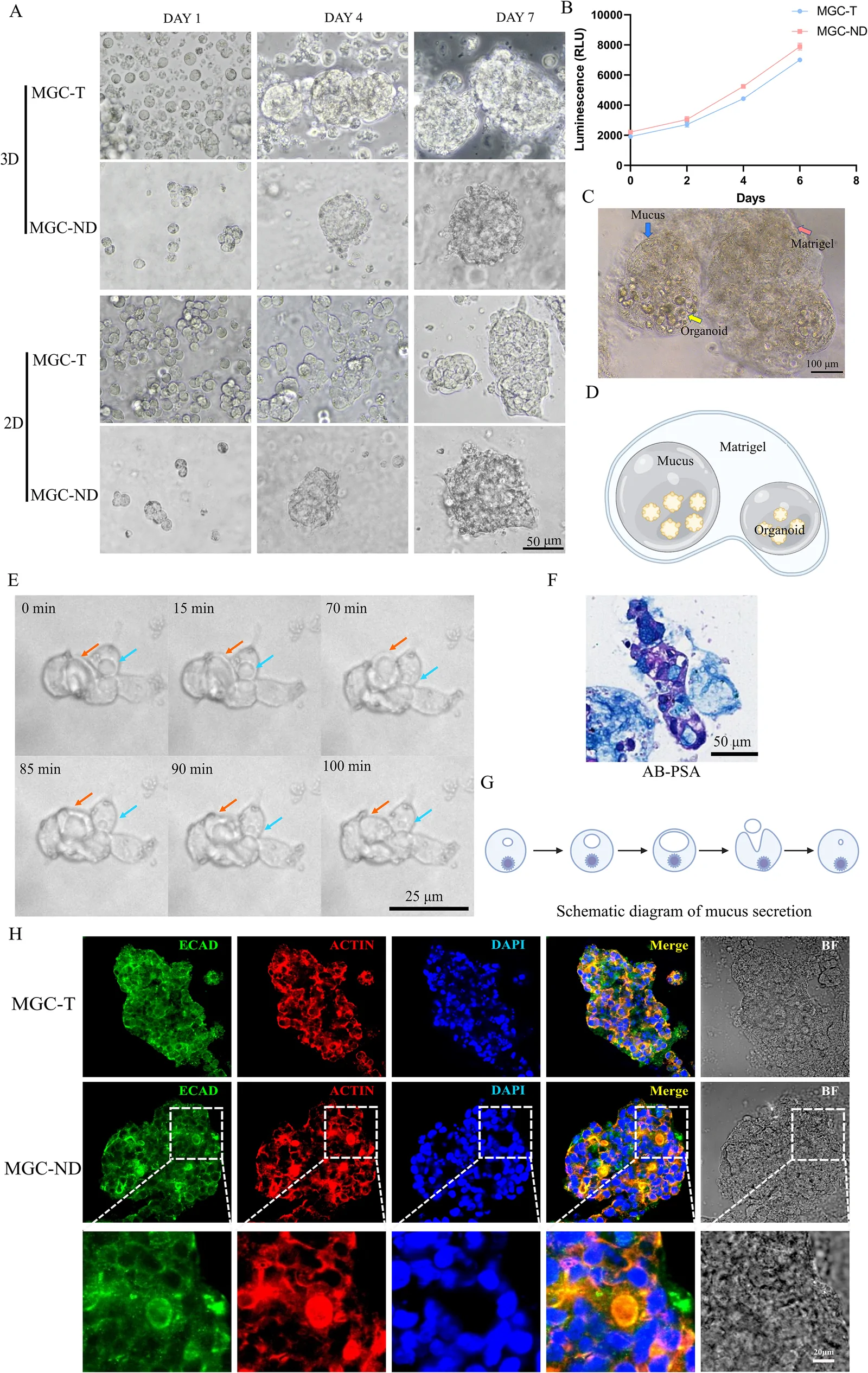
Characterization and Dynamic Analysis of Mucin Secretion in MGC-Derived Organoids. **(A)** Bright-field microscopy showing the morphological changes of MGC-T and MGC-ND organoids under three-dimensional culture conditions over 7 days. **(B)** Growth curves of MGC-T and MGC-ND organoids. Cell viability was assessed by CellTiter-Glo assay at the indicated time points. Data are presented as mean ± SD of three independent experiments. **(C)** Mucin secretion by MGC organoids in Matrigel as observed under light microscopy. Red arrows indicate mucin, green arrows indicate Matrigel, and yellow arrows indicate MGC organoids. Scale bar, 100 μm. **(D)** Schematic illustration of mucin secretion by MGC organoids in Matrigel. **(E)** Live-cell imaging capturing the dynamic process of mucin secretion by MGC-ND organoids, with orange and blue arrows highlighting the sequential changes within two mucin-containing vesicles. Scale bar, 25 µm. **(F)** Alcian Blue-Periodic Acid-Schiff (AB-PAS) staining for MGC organoids, illustrating mucin production. Scale bar, 50 µm. **(G)** Schematic diagram depicting the sequential stages of mucin secretion by MGC organoids. **(H)** Immunofluorescence staining of MGC organoids showing E-cadherin (ECAD, green) and F-actin (red), with nuclei counterstained with DAPI (blue). The magnified image highlights mucin localization. Scale bar, 20 µm.

A notable characteristic of these MGC organoids was their distinct mucin secretion capability. During cultivation, the accumulated mucin within the Matrigel gradually caused the organoids to detach from the matrix (**Figures 2C, D**). To further characterize the dynamic process of mucin secretion, we employed live cell imaging on MGC-ND organoids. Following enzymatic digestion and re-embedding in a thin layer of Matrigel, continuous 48-hour live-cell imaging revealed a dynamic sequence of events: a large, transparent vacuole gradually formed and expanded over approximately two hours, followed by a minor membrane rupture that initiated mucin release. Subsequently, the cell restored its normal morphology (**Figure 2E**). This observation was confirmed by positive AB-PAS staining results (**Figure 2F**). Notably, the fluorescence image clearly demonstrates mucin accumulation in areas devoid of DAPI staining (**Figure 2H**). The entire process was documented in a schematic illustration (**Figure 2G**), depicting the sequential stages of vacuole generation, expansion, and mucin secretion.

### 3.3 MGC organoids recapitulate the pathological and molecular characteristics of primary tumors

#### 3.3.1 Morphological features

Both MGC-T and MGC-ND organoids exhibited compact spheroid-like structures under bright-field microscopy (**Figure 3A**, left panel). HE staining revealed that organoids from both sources closely recapitulated the histological architecture of the corresponding parental tumors, characterized by pronounced nuclear heterogeneity and loss of cell polarity. Both organoids displayed glandular-like structures with abundant intracellular and extracellular mucin, mirroring the mucinous component of the primary tumor (**Figure 3A**, middle and right panels).

**Figure 3:**
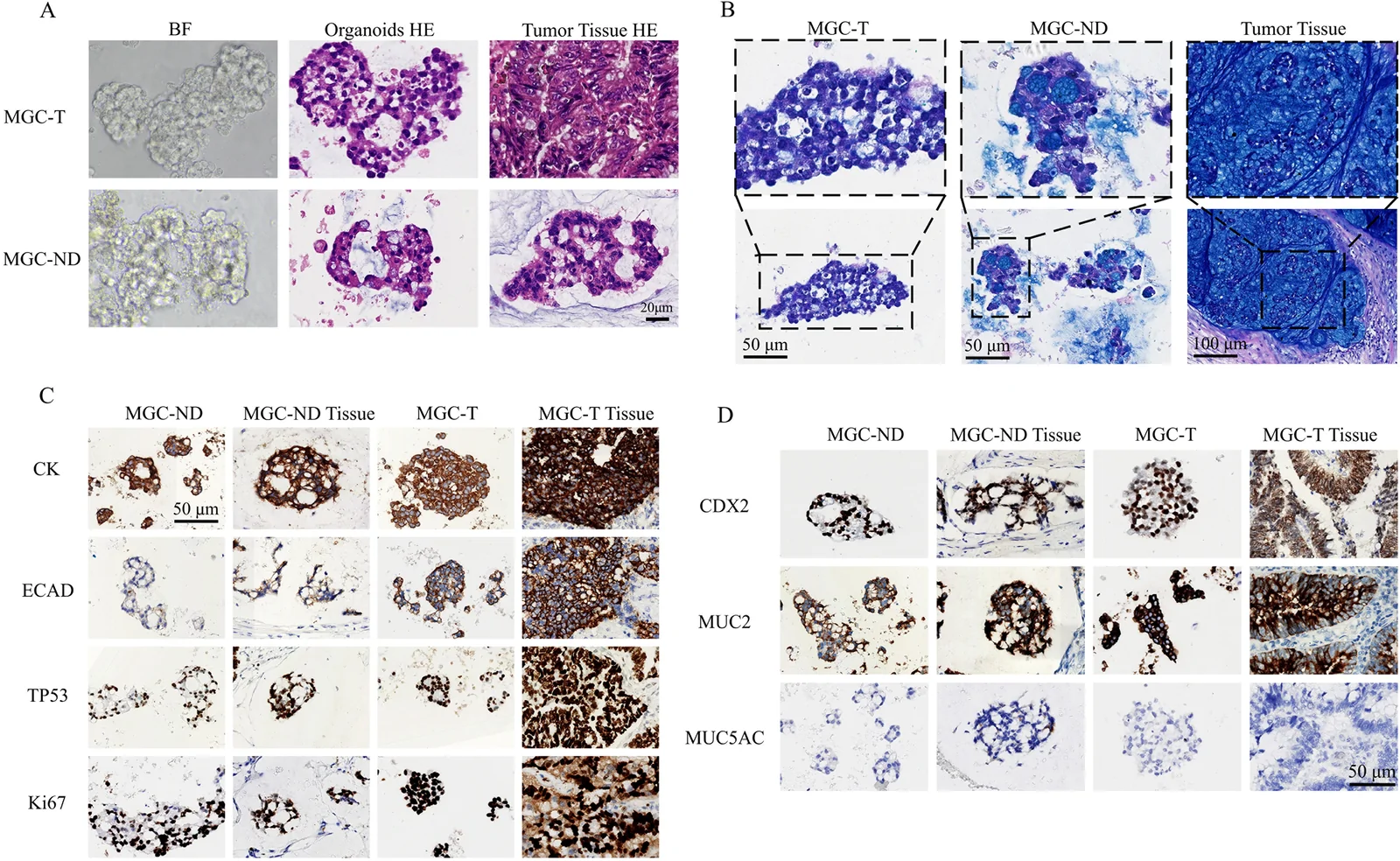
MGC organoids faithfully recapitulate the histopathological features of matched primary tissues. **(A)** HE staining for MGC organoids and matched tumor tissue. BF, Bright field; Scale bar, 20 μm. **(B)** Alcian Blue-Periodic Acid-Schiff (AB-PAS) staining for MGC organoids and matched tissues. Scale bar, 50 μm (organoids) and 100 μm (tissues). **(C)** IHC for epithelial and proliferation markers (CK, Ki67, p53, ECAD) in MGC organoids and matched tissues. Scale bar, 50 μm. **(D)** IHC for intestinal differentiation markers (CDX2, MUC2, MUC5AC) in MGC organoids and matched tissues. Scale bar, 50 μm.

#### 3.3.2 Mucin production

Alcian Blue staining confirmed robust mucin production in both MGC-T and MGC-ND organoids, comparable to that observed in their corresponding parental tissues (**Figure 3B**). This mucin accumulation was especially pronounced in the MGC-ND organoids, where extracellular vacuoles were most evident. Overall, these findings indicate that the MGC organoids faithfully retain the malignant features and mucin secretion characteristics of the parental tumor tissue.

#### 3.3.3 Immunohistochemical profiling

To characterize the molecular features of the organoids, we examined the expression of key cancer-related markers by immunohistochemistry. Both organoids and their matched tissues demonstrated strong cytoplasmic CK expression, confirming their epithelial origin. Robust nuclear Ki-67 staining indicated active proliferation, while strong nuclear TP53 positivity suggested abnormal p53 expression associated with poor prognosis. Notably, E-cadherin expression showed site-specific patterns, with high membrane expression in MGC-T but low expression in MGC-ND, matching their corresponding tissues and suggesting distinct adhesion properties (**Figure 3C**). Both MGC-T and MGC-ND, along with the matched tissues, expressed CDX2 and high levels of MUC2 while MUC5AC was absent (**Figure 3D**). This profile of high MUC2 expression coupled with MUC5AC loss supports an intestinal metaplastic origin of these gastric cancers.

### 3.4 High-throughput drug sensitivity screening reveals reproducible drug responses in MGC organoids

To investigate the drug sensitivity of MGC, we performed drug sensitivity screening on MGC organoids and six non-MGC gastric cancer organoids using 11 commonly used antitumor agents (**Figure 4**; **Supplementary Figure 1**). The pathological subtypes of the six non-MGC gastric cancer cases are detailed in **Supplementary Table 1**. The screening results revealed that MGC organoids exhibited relative resistance to first-line gastric cancer chemotherapeutic agents (oxaliplatin and 5-FU) compared with non-MGC organoids (15). Specifically, for 5-FU, the maximum screening concentration failed to reach the IC50 in MGC organoids. For oxaliplatin, the maximum screening concentration also did not reach the IC50 in MGC-T organoids, whereas MGC-ND organoids displayed an IC50 of 5.23 μM (**Figures 4A, B**), suggesting potential resistance to oxaliplatin in both lines. In contrast, taxane-based agents showed markedly higher sensitivity in MGC organoids relative to non-MGC organoids, with IC50 values of approximately 0.001 μM for paclitaxel and 0.0003 μM for docetaxel (**Figures 4A, B**). In clinical guidelines, these two drugs are typically recommended as second-line treatment options (16), raising the possibility that taxane-based chemotherapy may warrant further investigation in MGC. The drug sensitivity results for the other tested agents are presented in **Supplementary Figure 1** and **Supplementary Table 2**.

**Figure 4:**
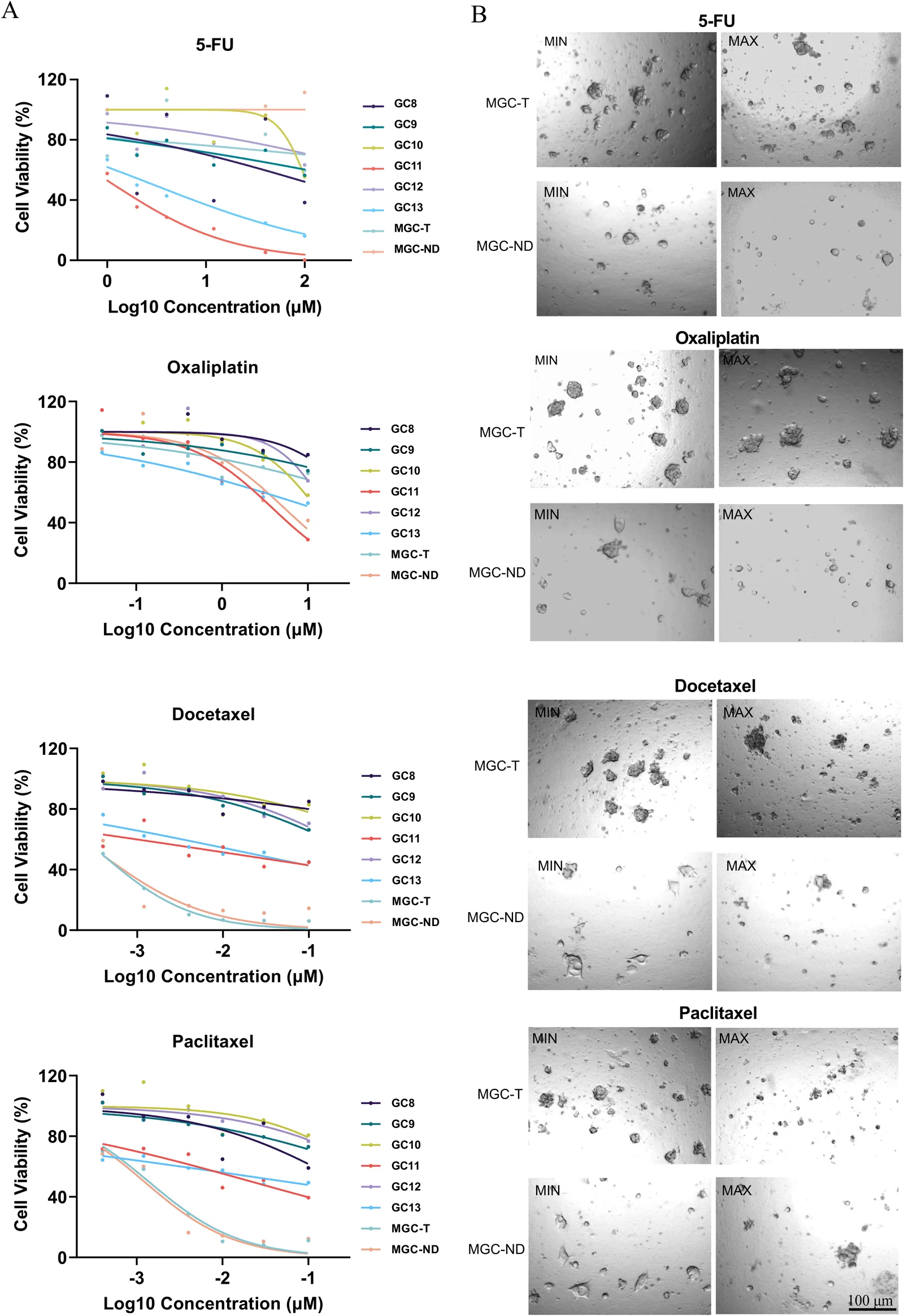
High-throughput Drug Sensitivity Testing of MGC-T and MGC-ND. **(A)** Dose-response curves for four commonly used chemotherapeutic drugs (5-fluorouracil [5-FU], oxaliplatin, docetaxel, and paclitaxel) were generated for both MGC-T and MGC-ND. Organoids were treated with serial concentrations of each drug for 72 h, and cell viability was measured by the CellTiter-Glo assay. Data are presented as mean ± SD from three independent experiments. **(B)** Representative bright-field images displaying the organoid morphology after *in vitro* treatment with each of the four drugs at the minimum (MIN) and maximum (MAX) concentrations. Scale bar, 100 µm.

To further validate our organoid-based drug sensitivity findings, we established two additional MGC organoid lines (designated MGC2 and MGC3) (**Figure 5A**). Consistently, AB-PAS staining revealed abundant extracellular mucus in both organoid lines and their corresponding tumor tissues (**Figure 5B**). Immunohistochemical analysis (pan-CK, E-cadherin, Ki-67, CDX2, MUC2 and MUC5AC) confirmed successful establishment of MGC organoids with retention of the molecular characteristics of the primary tumors (**Figures 5C, D**). We then performed single-agent drug sensitivity assays on these two MGC organoid lines using oxaliplatin, 5-FU, docetaxel and paclitaxel. Both MGC2 and MGC3 organoids exhibited resistance to oxaliplatin and 5-FU, with IC50 values exceeding the maximum screening concentrations. By contrast, the IC50 values for paclitaxel were 0.0011 μM and 0.0016 μM, respectively, and those for docetaxel were 0.0007 μM and 0.0005 μM, substantially lower than those observed in non-MGC organoids (**Figure 5E**; **Supplementary Table 2**).

**Figure 5:**
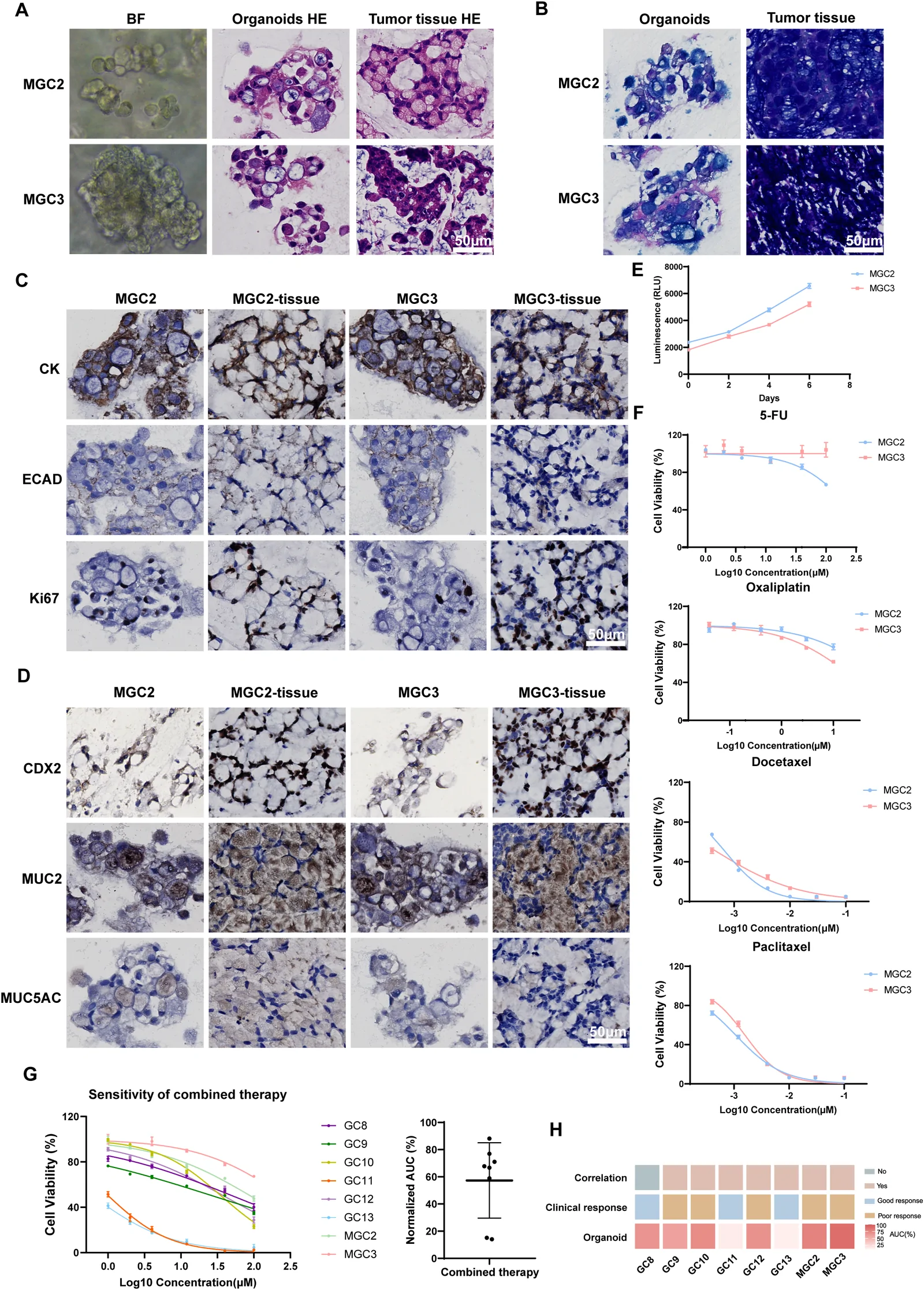
Establishment of additional MGC organoid lines and Clinical correlation of organoid-based drug sensitivity. **(A)** Bright-field and H&E staining of MGC2 and MGC3 organoids and their corresponding tumor tissues. Scale bars, 50 µm. **(B)** AB-PAS staining of MGC2 and MGC3 organoids and their corresponding tumor tissues. Scale bars, 50 µm. **(C)** IHC for conventional GC markers (CK, E-cadherin, Ki67) in MGC2 and MGC3 organoids and their corresponding tumor tissues. Scale bars, 50 µm. **(D)** IHC for intestinal-type GC markers (CDX2, MUC5AC, MUC2) in MGC2 and MGC3 organoids and their corresponding tumor tissues. Scale bars, 50 µm. **(E)** Growth curves of MGC2 and MGC3 organoids. Cell viability was assessed by CellTiter-Glo assay at the indicated time points. Data are presented as mean ± SD of three independent experiments. **(F)** Dose–response curves of MGC2 and MGC3 organoids to 5-FU, oxaliplatin, docetaxel and paclitaxel. Organoids were treated with serial concentrations of each drug for 72 h, and cell viability was measured by the CellTiter-Glo assay. Data are presented as mean ± SD of three independent experiments. **(G)** Dose-response curves of MGC2 and MGC3 organoids to combination treatment with oxaliplatin and 5-FU. The area under the curve (AUC) values derived from the dose-response data are presented as scatter plots (right panel). Data are shown as mean ± SD of three independent experiments. **(H)** Heatmap showing the correlation between organoid-based drug sensitivity and clinical chemotherapy responses in matched patients.

We further validated the organoid-based drug sensitivity and resistance profiles in patients receiving actual clinical treatment. A total of six non-MGC patients and two MGC patients received adjuvant chemotherapy based on oxaliplatin and 5-FU-containing regimens after surgery, with follow-up durations of 12–36 months. The MGC-T patient was lost to follow-up and was therefore excluded. Patients who experienced radiological recurrence within 6 months after surgery were classified as poor responders to chemotherapy, consistent with the typical window for early recurrence detection in gastric cancer patients receiving adjuvant chemotherapy. To better mirror the clinical practice, we performed combination drug sensitivity assays using oxaliplatin plus 5-FU on organoids derived from these eight patients and calculated the area under the curve (AUC) (**Figures 5F, G**). Organoids with an AUC ≥ 50% were defined as resistant, whereas those with an AUC < 50% were defined as sensitive (17). Among the eight patients, the organoid-based drug sensitivity results were consistent with the clinical response in seven patients (87.5%) (**Figure 5H**; **Supplementary Table 3**). For example, patient GC8 received adjuvant chemotherapy with oxaliplatin and S-1 and remained recurrence-free during 36 months of follow-up, and the corresponding organoids demonstrated sensitivity to the combination of oxaliplatin and 5-FU. In contrast, patient MGC2 experienced tumor recurrence detected by computed tomography at 5 months post-surgery, and the organoids exhibited resistance to the oxaliplatin and 5-FU combination. These findings preliminarily validate the reliability of our organoid-based drug sensitivity testing and suggest that patient-derived gastric cancer organoids may serve as a useful tool for drug screening and for predicting individual patient responses to chemotherapy.

## 4 Discussion

MGC represents a rare and aggressive histological subtype of gastric adenocarcinoma, characterized by abundant extracellular mucin pools that account for more than 50% of the tumor volume (18). Despite its distinct pathological features and poor prognosis (4, 19), MGC remains understudied due to the lack of representative preclinical models that faithfully recapitulate its unique biological behaviors. Compared with immortalized gastric cancer cell lines and patient-derived xenograft models (20), organoids have emerged as a powerful research tool because they preserve the complex phenotypic heterogeneity of the original tumors (21, 22). In this study, we successfully established patient-derived MGC organoids from three MGC patients, including paired primary and metastatic lines (MGC-T, MGC-ND) from one patient and two additional independent MGC lines (MGC2, MGC3). These organoids not only retained the histopathological and molecular characteristics of the parental tumors but also exhibited a reproducible drug sensitivity pattern distinct from that of non-MGC gastric cancer organoids, particularly demonstrating marked sensitivity to taxane-based agents while showing resistance to standard first-line chemotherapy. These findings highlight the potential of MGC organoids as a robust preclinical platform for investigating tumor heterogeneity, mucin secretion dynamics, and personalized therapeutic strategies.

The successful establishment of patient-derived organoids from MGC tissues presented unique technical challenges due to the abundant mucin-rich extracellular matrix, which interfered with standard tissue digestion protocols and organoid embedding procedures. In this study, we optimized the enzymatic digestion and culture conditions to achieve robust organoid growth from both the primary tumor and peritoneal metastatic lesions. Both MGC-T and MGC-ND organoids exhibited stable growth kinetics and could be maintained through multiple passages, demonstrating the feasibility of MGC organoid culture when appropriate technical modifications are applied. Our methodological approach provides a reproducible framework for future MGC organoid establishment, potentially facilitating the development of larger MGC-specific organoid biobanks for translational research.

Comprehensive histological and molecular validation confirmed that all four MGC organoid lines (MGC-T, MGC-ND, MGC2 and MGC3) faithfully retained the malignant features of their corresponding parental tissues. HE staining revealed the characteristic glandular architecture with abundant intracellular and extracellular mucin, while Alcian Blue staining confirmed robust acidic mucin production—a hallmark feature of MGC. Immunohistochemical profiling further demonstrated conservation of key molecular markers including cytokeratin, E-cadherin, and Ki-67, as well as the intestinal differentiation markers CDX2 and MUC2. Paired primary and metastatic organoids (MGC-T and MGC-ND) additionally showed TP53 expression patterns consistent with their parental tumors. These validation results align with the growing body of evidence demonstrating that gastric cancer organoids maintain the histological architecture and protein expression profiles of the original tumors (17, 23). The dynamic mucin secretion process observed in our MGC organoids represented a distinctive biological feature with potential clinical relevance. Live-cell imaging captured the sequential stages of intracellular vacuole formation, expansion, and eventual mucin release through transient membrane rupture, followed by rapid cellular recovery. This observation provides direct visual evidence of the active mucin secretion mechanism in MGC, which likely contributes to the characteristic mucin pools observed in patient tumors.

The accumulation of secreted mucin within the Matrigel matrix gradually caused organoid detachment, a phenomenon that had important implications for organoid culture maintenance and likely reflects the *in vivo* behavior of MGC cells in the peritoneal cavity. Excessive mucin production in MGC patients can result in massive mucin accumulation within the peritoneal cavity, a condition reminiscent of pseudomyxoma peritonei that may cause bowel obstruction and therapeutic challenges (24). Our organoid model offers a unique platform to investigate the cellular and molecular mechanisms underlying mucin hypersecretion, as well as to test potential therapeutic interventions targeting mucin production or clearance.

The high-throughput drug sensitivity screening conducted on our MGC organoids yielded clinically significant findings with potential therapeutic implications. All four MGC organoid lines (MGC-T, MGC-ND, MGC2 and MGC3) exhibited marked resistance to 5-FU and oxaliplatin, the backbone of standard first-line chemotherapy regimens for gastric cancer (15). This resistance is likely attributed to the intrinsic properties of MGC as a histologically distinct subtype, which is naturally less sensitive to DNA-damage cytotoxic agents (25). Moreover, elevated MUC2 expression, the predominant mucin in MGC, appears to further enhance its resistance to these agents (26).

In contrast, all MGC organoids demonstrated exceptional sensitivity to taxane-based agents, with paclitaxel and docetaxel showing IC50 values in the nanomolar to sub-nanomolar range. These findings align with current clinical guidelines and recent evidence supporting taxane-based regimens as effective second-line treatments for advanced gastric cancer (15, 16). These preclinical findings may provide a mechanistic explanation for recent clinical observations that mucinous gastric cancer achieves favorable histopathological response to taxane-containing perioperative chemotherapy regimens such as FLOT (27). We speculate that the taxane component of FLOT may be particularly critical for MGC, as these mucin-secreting cells with complex cellular and tissue architecture involved in mucin discharge rely on microtubule dynamics to carry out essential biological functions—the precise targets of taxane drugs.

The discordance between first-line resistance and second-line sensitivity observed in our MGC organoids highlights the critical importance of functional drug testing in guiding personalized treatment selection. Previous studies have reported high concordance rates exceeding 90% between organoid predictions and actual patient responses, establishing patient-derived organoids as reliable tools for precision oncology (17, 28). To validate the correlation between our *in vitro* findings and clinical outcomes, we analyzed the combined oxaliplatin and 5-FU drug sensitivity data from eight patient-derived organoids alongside the corresponding clinical chemotherapy responses. Encouragingly, the organoid-based sensitivity profiles were consistent with clinical responses in seven of the eight cases (87.5%), providing preliminary evidence supporting the predictive potential of organoid-based drug testing in this context. In the context of MGC, where treatment options are limited and prognosis is poor, organoid-guided therapeutic selection could potentially identify effective regimens for individual patients, avoiding unnecessary toxicity from ineffective first-line treatments.

Nevertheless, several limitations should be considered when interpreting the present findings. First, although we successfully established three MGC organoid lines from independent patients, the sample size remains small (n=3), and the generalizability of our observations to the broader MGC population requires validation in larger, multicenter cohorts. Second, the clinical correlation analysis was retrospective and limited by a small patient cohort, precluding definitive conclusions regarding the predictive utility of organoid-based drug testing. Third, while our data indicate a consistent pattern of taxane sensitivity in MGC organoids, the underlying molecular mechanisms remain unexplored. Future comparative transcriptomic or proteomic profiling between taxane-sensitive and taxane-resistant lines may help identify predictive biomarkers and inform patient stratification in prospective trials. Overall, larger prospective studies incorporating clinically relevant combination regimens and systematic longitudinal follow-up are warranted to fully establish the clinical utility of organoid-guided therapeutic selection in MGC.

It is also important to acknowledge a technical limitation of our drug sensitivity assay. We employed the standard CellTiter-Glo reagent, which is optimized for 2D monolayer cultures. For 3D hydrogel-embedded organoids, particularly those with abundant mucin production as seen in MGC, this method may suffer from suboptimal reagent penetration and incomplete ATP release due to the physical barriers imposed by the extracellular matrix and secreted mucus. Such limitations could introduce non-uniform lysis efficiency across organoids of varying sizes and mucin content, potentially affecting the accuracy of viability quantification and contributing to apparent resistance patterns. Our findings should therefore be interpreted as qualitative trends, and future studies employing 3D-optimized viability assays (e.g., CellTiter-Glo 3D) are warranted for quantitative validation.

## 5 Conclusions

In conclusion, we have successfully established and characterized patient-derived organoid models of mucinous gastric cancer, demonstrating their faithful retention of histopathological, molecular, and functional characteristics of the parental tumors. The reproducible drug sensitivity pattern observed—particularly the resistance to standard first-line agents (5-FU and oxaliplatin) and marked sensitivity to taxane-based drugs (paclitaxel and docetaxel)—highlights the potential clinical utility of MGC organoids in guiding personalized treatment selection. The detailed characterization of mucin secretion dynamics provides novel insights into the biology of this aggressive gastric cancer subtype. While further validation in larger patient cohorts is warranted, our study establishes a valuable platform for future translational research in MGC and underscores the broader value of subtype-specific organoid models in advancing precision oncology for gastric cancer.

## Data Availability

The original contributions presented in the study are included in the article/**Supplementary Material**. Further inquiries can be directed to the corresponding authors.

## Supplementary material

Supplementary Figure 1 Dose–response curves for 7 chemotherapeutic agents were evaluated in both MGC-T and MGC-ND.

## References

[1] Bray F Laversanne M Sung H Ferlay J Siegel RL Soerjomataram I . Global cancer statistics 2022: GLOBOCAN estimates of incidence and mortality worldwide for 36 cancers in 185 countries. CA Cancer J Clin. (2024) 74:229–63. doi: 10.3322/caac.21834 38572751

[2] Hu B El Hajj N Sittler S Lammert N Barnes R Meloni-Ehrig A . Gastric cancer: Classification, histology and application of molecular pathology. J Gastrointest Oncol. (2012) 3:251–61. doi: 10.3978/j.issn.2078-6891.2012.021 PMC3418539 22943016

[3] Kunisaki C Akiyama H Nomura M Matsuda G Otsuka Y Ono HA . Clinicopathologic characteristics and surgical outcomes of mucinous gastric carcinoma. Ann Surg Oncol. (2006) 13:836–42. doi: 10.1245/aso.2006.03.077 16604474

[4] Ma Y Zhang S Wang Y Hu C Chen J Pang C . Comparison of clinicopathological features and prognosis of mucinous gastric carcinoma and other gastric cancers: A retrospective study of 4,417 patients. J Gastrointest Surg. (2023) 27:2352–64. doi: 10.1007/s11605-023-05853-z 37848685

[5] Park YS Kook MC Kim BH Lee HS Kang DW Gu MJ . A standardized pathology report for gastric cancer: 2nd edition. J Gastric Cancer. (2023) 23:107–45. doi: 10.4132/jptm.2022.12.23 36750994 PMC9911618

[6] Choi JS Kim MA Lee HE Lee HS Kim WH . Mucinous gastric carcinomas: clinicopathologic and molecular analyses. Cancer. (2009) 115:3581–90. doi: 10.1002/cncr.24422 19479974

[7] Tajima Y Shimoda T Nakanishi Y Yokoyama N Tanaka T Shimizu K . Gastric and intestinal phenotypic marker expression in gastric carcinomas and its prognostic significance: immunohistochemical analysis of 136 lesions. Oncology. (2001) 61:212–20. doi: 10.1159/000055377 11574777

[8] Song K Yang Q Yan Y Yu X Xu K Xu J . Gastric mucin phenotype indicates aggressive biological behaviour in early differentiated gastric adenocarcinomas following endoscopic treatment. Diagn Pathol. (2021) 16:62. doi: 10.1186/s13000-021-01122-2 34256780 PMC8276406

[9] Tan P Yeoh KG . Genetics and molecular pathogenesis of gastric adenocarcinoma. Gastroenterology. (2015) 149:1153–1162.e3. doi: 10.1053/j.gastro.2015.05.059 26073375

[10] Giroux V Rustgi AK . Metaplasia: tissue injury adaptation and a precursor to the dysplasia-cancer sequence. Nat Rev Cancer. (2017) 17:594–604. doi: 10.1038/nrc.2017.68 28860646 PMC5998678

[11] Dinis-Ribeiro M Kuipers EJ . How to manage a patient with gastric intestinal metaplasia: An international perspective. Gastroenterology. (2020) 158:1534–7. doi: 10.1053/j.gastro.2020.01.008 31926996

[12] Reis CA David L Correa P Carneiro F de Bolós C Garcia E . Intestinal metaplasia of human stomach displays distinct patterns of mucin (MUC1, MUC2, MUC5AC, and MUC6) expression. Cancer Res. (1999) 59:1003–7. 10070955

[13] Tan T Mouradov D Gibbs P Sieber OM . Protocol for generation of and high-throughput drug testing with patient-derived colorectal cancer organoids. STAR Protoc. (2024) 5:103090. doi: 10.1016/j.xpro.2024.103090 38809757 PMC11166870

[14] Amin MB Greene FL Edge SB Compton CC Gershenwald JE Brookland RK . The eighth edition AJCC cancer staging manual: Continuing to build a bridge from a population-based to a more "personalized" approach to cancer staging. CA Cancer J Clin. (2017) 67:93–9. doi: 10.3322/caac.21388 28094848

[15] Ajani JA D'Amico TA Bentrem DJ Corvera CU Das P Enzinger PC . Gastric cancer, version 2.2025, NCCN clinical practice guidelines in oncology. J Natl Compr Canc Netw. (2025) 23:169–91. doi: 10.6004/jnccn.2025.0022 40341199

[16] Ford HE Marshall A Bridgewater JA Janowitz T Coxon FY Wadsley J . Docetaxel versus active symptom control for refractory oesophagogastric adenocarcinoma (COUGAR-02): an open-label, phase 3 randomised controlled trial. Lancet Oncol. (2014) 15:78–86. doi: 10.1016/s1470-2045(13)70549-7 24332238

[17] Zhao Y Li S Zhu L Huang M Xie Y Song X . Personalized drug screening using patient-derived organoid and its clinical relevance in gastric cancer. Cell Rep Med. (2024) 5:101627. doi: 10.1016/j.xcrm.2024.101627 38964315 PMC11293329

[18] Nagtegaal ID Odze RD Klimstra D Paradis V Rugge M Schirmacher P . The 2019 WHO classification of tumours of the digestive system. Histopathology. (2020) 76:182–8. doi: 10.1111/his.13975 31433515 PMC7003895

[19] Choi MG Sung CO Noh JH Kim KM Sohn TS Kim S . Mucinous gastric cancer presents with more advanced tumor stage and weaker β-catenin expression than nonmucinous cancer. Ann Surg Oncol. (2010) 17:3053–8. doi: 10.1245/s10434-010-1184-z 20645013

[20] Choi YY Lee JE Kim H Sim MH Kim KK Lee G . Establishment and characterisation of patient-derived xenografts as paraclinical models for gastric cancer. Sci Rep. (2016) 6:22172. doi: 10.1038/srep22172 26926953 PMC4772087

[21] Yan HHN Siu HC Law S Ho SL Yue SSK Tsui WY . A comprehensive human gastric cancer organoid biobank captures tumor subtype heterogeneity and enables therapeutic screening. Cell Stem Cell. (2018) 23:882–897.e11. doi: 10.1016/j.stem.2018.09.016 30344100

[22] Ling R Wu Y Yan Z Xian Q Chu Y Zhu W . Organoid models: applications and research advances in gastric cancer. Front Oncol. (2026) 16:1766571. doi: 10.3389/fonc.2026.1766571 42137168 PMC13169162

[23] Xu J Yu B Wang F Yang J . Xenograft and organoid models in developing precision medicine for gastric cancer (Review). Int J Oncol. (2024) 64:41. doi: 10.3892/ijo.2024.5629 38390969 PMC10919760

[24] Morera-Ocon FJ Navarro-Campoy C . History of pseudomyxoma peritonei from its origin to the first decades of the twenty-first century. World J Gastrointest Surg. (2019) 11:358–64. doi: 10.4240/wjgs.v11.i9.358 31572561 PMC6766476

[25] Tajima Y Shimada Y Kameyama H Yagi R Okamura T Kobayashi T . Association between poorly differentiated clusters and efficacy of 5-fluorouracil-based adjuvant chemotherapy in stage III colorectal cancer. Jpn J Clin Oncol. (2017) 47:313–20. doi: 10.1093/jjco/hyw209 28100684 PMC5444335

[26] Behzatoğlu K . The power of MUC2 in mucinous adenocarcinomas. Pathobiology. (2026) 93(4):232–45. doi: 10.1159/000550592 PMC12995350 41557567

[27] Caspers IA Slagter AE Vissers PAJ Lopez-Yurda M Beerepoot LV Ruurda JP . Histopathological response to chemotherapy and survival of mucinous type gastric cancer. J Natl Cancer Inst. (2025) 117:253–61. doi: 10.1093/jnci/djae227 39276158 PMC11807439

[28] Schmäche T Fohgrub J Klimova A Laaber K Drukewitz S Merboth F . Stratifying esophago-gastric cancer treatment using a patient-derived organoid-based threshold. Mol Cancer. (2024) 23:10. doi: 10.1186/s12943-023-01919-3 PMC10777586 38200602

