# Supplementary material for: Establishment and characterization of patient-derived mucinous gastric cancer organoids with faithful histopathological features and reproducible drug response patterns
Source: Front Oncol. 2026 Sep 17;16:1905751. doi: 10.3389/fonc.2026.1905751 (PMC13626893; doi:10.3389/fonc.2026.1905751)
Supplement: Supplementary file 1 [file Table2.docx]

Supplementary Table 2. Drug screening of patient-derived organoids to chemotherapeutics, related to Figure 4-5 and Supplementary Figure 1.

| Replicate 1 | IC50 | GC8 | GC9 | GC10 | GC11 | GC12 | GC13 | MGC-T | MGC-ND | MGC2 | MGC3 |
| --- | --- | --- | --- | --- | --- | --- | --- | --- | --- | --- | --- |
|  | 5-FU(µM) | 129.30 | 126.70 | 108.00 | >100.00 | 296.20 | 3.00 | 383902.00 | >100.00 | 199.30 | >100.00 |
|  | Oxaliplatin(μM) | 43.59 | 325.00 | 12.50 | 3.80 | 16.19 | 11.06 | 111.50 | 5.23 | 64.30 | 19.05 |
|  | Docetaxel(μM) | 44.78 | 0.38 | 1.75 | 0.01 | 0.41 | 0.02 | 0.0004 | 0.0004 | 0.0007 | 0.0005 |
|  | Paclitaxel(μM) | 0.250 | 1.262 | 0.678 | 0.022 | 1.102 | 0.055 | 0.001 | 0.001 | 0.001 | 0.002 |
|  | Epirubicin(μM) | 2.944 | 1.560 | 1.163 | 0.437 | 2.151 | 0.268 | 0.017 | 0.003 | - | - |
|  | Doxorubicin(μM) | 2.605 | 1.097 | 1.105 | 0.003 | 1.951 | 0.296 | 0.156 | 0.024 | - | - |
|  | Cisplatin(μM) | 38.810 | >33.000 | 33.600 | 11.870 | 96.110 | 9.707 | 7.281 | 0.002 | - | - |
|  | Alpelisib(μM) | 18.580 | 5846.000 | 15.650 | 3.660 | >10.000 | 1.169 | 2.455 | 1.223 | - | - |
|  | Lapatinib(μM) | 5.301 | 1.891 | 3.148 | 1.746 | >10.000 | 0.269 | 0.627 | 0.884 | - | - |
|  | Apatinib(μM) | 11.67 | 565.90 | 9.93 | 3.35 | 15.26 | 358345.00 | 8.47 | 7.74 | - | - |
|  | Sunitinib(μM) | 3.02 | 6.91 | 2.97 | 2.42 | >10.000 | 4.92 | 3.72 | 3.14 | - | - |
|  |  |  |  |  |  |  |  |  |  |  |  |
| Combined therapy |  | GC8 | GC9 | GC10 | GC11 | GC12 | GC13 | MGC2 | MGC3 |  |  |
|  | IC50 (μM) | 51.24 | 27.36 | 35.60 | 1.01 | 38.64 | 0.61 | 90.04 | 253.70 |  |  |
|  | AUC (%) | 66.70 | 58.90 | 71.05 | 15.10 | 67.75 | 14.04 | 76.95 | 88.10 |  |  |
