# Supplementary material for: Establishment and characterization of patient-derived mucinous gastric cancer organoids with faithful histopathological features and reproducible drug response patterns
Source: Front Oncol. 2026 Sep 17;16:1905751. doi: 10.3389/fonc.2026.1905751 (PMC13626893; doi:10.3389/fonc.2026.1905751)
Supplement: Supplementary file 2 [file Table3.docx]

**Supplementary Table 3. Drug responses in organoid corresponding follow-up gastric cancer patients, related to Figure 5.**

| **Number** | **Metastasis** | **Therapy prior to derivation** | **Postoperative Chemotherapy** | **Clinical response** | **Organoid treatment** | | **Organoid response** |
| --- | --- | --- | --- | --- | --- | --- | --- |
|  |  |  |  |  | **IC50 (μM)** | **AUC (%)** |  |
| MGC2 | None | None | SOX (S-1+Oxaliplatin) *5 | Recurrence and metastasis (5 months) | 90.04 | 76.95 | resistant |
| MGC3 | None | None | FOLFOX (Oxaliplatin+5-FU) *7 | Recurrence (5 months) | 253.7 | 88.10 | resistant |
| GC8 | None | None | SOX (S-1+Oxaliplatin) *4 | No recurrence (30 months) | 51.24 | 66.70 | resistant |
| GC9 | None | None | SOX (S-1+Oxaliplatin) *4 | Recurrence (6 months) | 27.36 | 58.90 | resistant |
| GC10 | None | None | SOX (S-1+Oxaliplatin) *6 | Recurrence (5 months) | 35.60 | 71.05 | resistant |
| GC11 | None | None | FOLFOX (Oxaliplatin+5-FU) *5 | No recurrence (28 months) | 1.01 | 15.10 | sensitive |
| GC12 | None | None | SOX (S-1+Oxaliplatin) *3 | Recurrence (6 months) | 38.64 | 67.75 | resistant |
| GC13 | None | None | FOLFOX (Oxaliplatin+5-FU) *7 | No recurrence (15 months) | 0.61 | 14.04 | sensitive |
